# Behavioral changes preceded by subthalamic nucleus activity alterations in a progressive macaque model of Parkinson’s disease

**DOI:** 10.1038/s41531-026-01498-2

**Published:** 2026-07-31

**Authors:** Mathilde Bertrand, Stephan Chabardes, Jessy Hugues Dit Ciles, Nicolas De Leiris, Julien Bastin, Brigitte Piallat

**Affiliations:** 1 Inserm, U1216, Grenoble Institute of Neurosciences, Université Grenoble Alpes, Grenoble, France; 2 Department of Neurosurgery, Inserm, U1216, CHU Grenoble Alpes, Grenoble Institute Neurosciences, Université Grenoble Alpes, Grenoble, France; 3 Clinatec-CEA Leti, Grenoble, France; 4 Service de Médecine Nucléaire, INSERM, CHU Grenoble Alpes, LRB, Université Grenoble Alpes, Grenoble, France

**Keywords:** Neurology, Neuroscience

## Abstract

Parkinson’s disease (PD) is diagnosed after motor symptoms appear, although non-motor symptoms emerge years earlier. Following years of pharmacological treatment, high-frequency stimulation (HFS) of the subthalamic nucleus (STN), a key hub in goal-directed behaviors, can be proposed. While HFS-STN reliably improves motor symptoms, it does not specifically address non-motor symptoms. Clarifying how STN dysfunction contributes to these symptoms could improve stimulation strategies. Here, we longitudinally recorded STN local field potentials in two macaques performing a demanding task during chronic low-dose MPTP treatment. This progressive model, evolving from an asymptomatic stage to motivational, cognitive, and motor deficits, enabled detailed characterization of non-motor stages preceding motor impairment. Each stage was associated with distinct electrophysiological alterations, including early loss of reward-related theta activity, followed by disappearance of decision-related theta oscillations and later reduction of movement-related beta rebound. In the stable parkinsonian stage, stimulation of different STN territories produced complementary behavioral effects: dorsal HFS improved motor performance, whereas ventral low-frequency stimulation alleviated motivational deficits. These findings reveal a temporal relationship between STN dysfunction and symptom onset and support site- and frequency-specific stimulation strategies to address motor and non-motor symptoms in PD.

## Introduction

Parkinson’s disease (PD) is a progressive neurodegenerative disorder characterized not only by cardinal motor symptoms but also by a range of non-motor symptoms, including sleep/wake disturbances, apathy, and cognitive decline, which precede motor signs by years^1–3^. Understanding the neural substrates of these early manifestations is critical for enabling early diagnosis and developing targeted therapies^4^. The subthalamic nucleus (STN) is a central hub in the basal ganglia-thalamo-cortical network, integrating cortical inputs to shape and adapt goal-directed behaviors^5,6^. In PD, pathological STN beta-band activity (12–35 Hz) is associated with motor symptoms occurrence and abolished by dorsolateral STN high-frequency stimulation (HFS, ~130 Hz), which improves motor symptoms^7–10^. Beyond motor control, accumulating evidence implicates STN theta-band (4–8 Hz) alterations with non-motor PD symptoms, including impaired conflict resolution, apathy, impulsivity, and depression^11–15^.

We recently demonstrated correlations between physiological STN activity and distinct behavioral domains in non-human primates (NHPs), providing a framework for studying how these dynamics progress during PD^16^. Among available preclinical models, the NHP parkinsonian model induced by 1-methyl-4-phenyl-1,2,3,6-tetrahydropyridine (MPTP) remains the gold standard, as it reproduces both motor and non-motor impairments alongside nigro-striatal degeneration^17–19^. In particular, chronic low-dose MPTP treatment induces a progressive PD phenotype^20,21^ enabling longitudinal study of STN activity and its temporal relationship to the onset of non-motor and motor manifestations.

Deep brain stimulation (DBS) provides a causal approach to explore STN functions. While HFS of the dorsolateral STN reliably alleviates motor symptoms, it can exacerbate non-motor impairments^22,23^. Conversely, dorsal low-frequency stimulation (LFS, ~4–5 Hz) has shown promise for improving non-motor symptoms in PD patients, including deficits in conflict resolution and impulsivity, likely by engaging prefrontal-STN hyperdirect pathway^24–26^. With emerging adaptive DBS technologies enabling site and frequency-specific modulation^27–30^, clarifying the temporal and functional dynamics of STN activity alterations has become a pressing clinical priority.

Here, we investigated the longitudinal relationship between STN activity and the sequential emergence of motivational, cognitive, and motor deficits in a progressive NHP model of PD. We reveal that STN activity alterations precede behavioral changes, with theta disruptions related to motivational and cognitive deficits, and abnormal beta activity associated with motor symptoms. In a stable parkinsonian syndrome, we further demonstrate site and frequency-specific STN stimulation effects, with dorsal HFS improving motor impairments and ventral LFS alleviating motivational deficits.

## Results

### Progressive parkinsonian syndrome

**Fig. 1:**
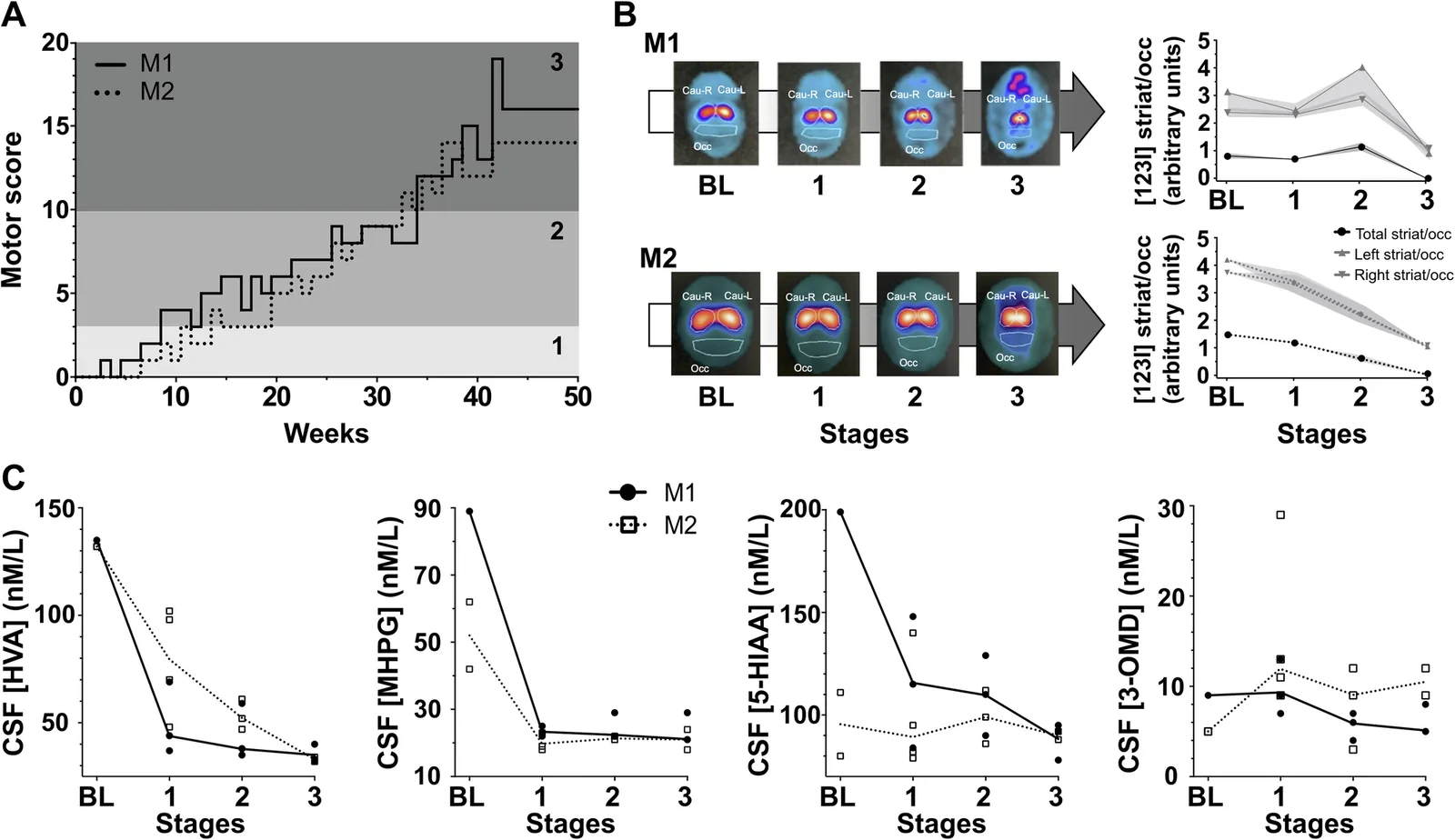
Progressive parkinsonian syndrome for M1 (solid line) and M2 (dotted line). **A** Weekly evaluation of the parkinsonian syndrome induced by chronic low-dose MPTP administration. Motor score discriminated 3 stages: 1-asymptomatic (very light gray, score 0–3); 2-mild parkinsonism (light gray, score 4–10); 3-moderate parkinsonism (dark gray, score >10). **B** DaT SPECT imaging using Ioflupane (I^123^) tracer for each stage with ratios quantification between the left and right striatum (Cau-L and Cau-R) relative to cerebral background noise (Occ). **C** Lumbar cerebrospinal fluid (CSF) concentrations of homovanillic acid (HVA), 5-hydroxyindole acetic acid (5-HIAA), 3-methoxy-4-hydroxyphenylglycol (MHPG), and 3-ortho-methyldopa (3-OMD). BL, baseline before MPTP treatment.

Chronic low-dose MPTP administration (0.2–0.5 mg/kg, biweekly) induced a progressive parkinsonian syndrome in both monkeys. M1 received 20 injections (5.45 mg/kg over 43 weeks), and M2 received 18 injections (6.7 mg/kg over 39 weeks), and both reached a similar stable moderate parkinsonian syndrome. Using a validated scale to evaluate motor manifestations^31^, we identified three severity stages (Fig. 1A): stage 1 (score 0–3), asymptomatic, with no motor signs; stage 2 (score 4–10), mild parkinsonism, with light and unstable deficits; and stage 3 (score >10), moderate parkinsonism, with significant and stable motor manifestations, including reduced activity, bradykinesia, and flexed posture.

Longitudinal DaT SPECT imaging revealed a progressive decrease in striatal Ioflupane tracer relative to the occipital cortex, beginning after the first MPTP injection (Fig. 1B). Both monkeys developed similar trajectories, except for a transient increase during stage 2 for M1. This observation prompted a deeper behavioral analysis, which led us to define an additional stage (stage 2’).

Finally, we analyzed serial cerebrospinal fluid (CSF) to identify metabolite correlates of the dopaminergic deficit (Fig. 1C). In both monkeys, homovanillic acid (HVA), principal dopamine metabolite, progressively decreased, and 3-methoxy-4-hydroxyphenylglycol (MHPG), major noradrenaline metabolite, was significantly reduced from stage 1. M1 also showed a progressive decrease in 5-hydroxy indoleacetic acid (5-HIAA), main serotonin metabolite, whereas 3-ortho-methyldopa (3-OMD), product of dopamine synthesis, remained unchanged.

### Early non-motor manifestations

**Fig. 2:**
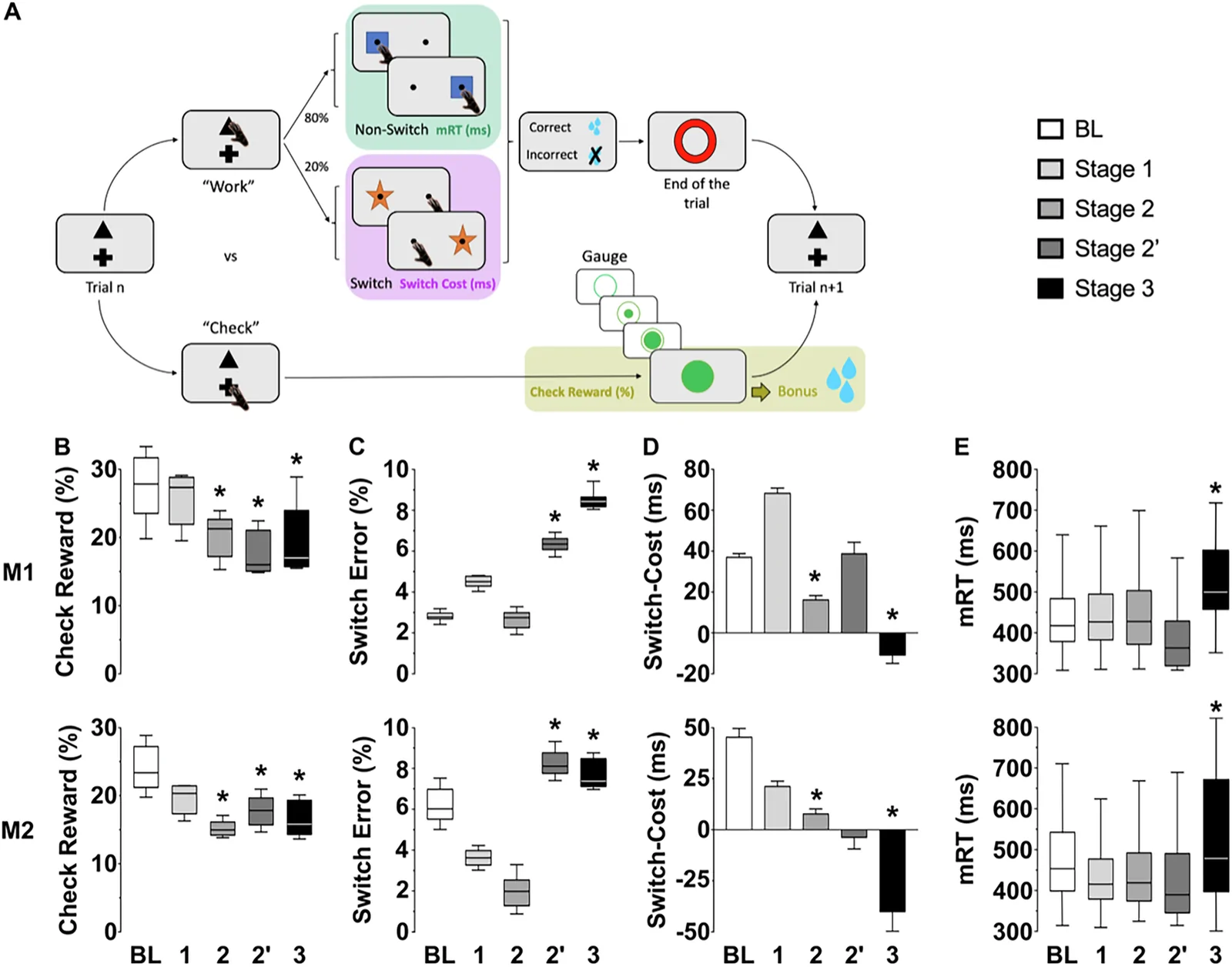
Behavioral effect of MPTP treatment across stages. **A** Rewarded task assessing limbic (gold), cognitive (pink), and motor (green) behaviors. On each trial, monkeys decided to “Work” on the main switching task, or “Check” the size of a gauge indicating the proximity of a bonus reward (see “Methods”). **B** Probabilities to check when the reward is available (Check Reward, %). **C** Errors on Switch trials (Switch Error, %). **D** Switch-Cost in ms, response time difference between Switch and Non-Switch trials. **E** Motor response time (mRT, ms) on Non-Switch trials. Stages: baseline, before MPTP treatment (BL, white); stage 1, asymptomatic (1, very light gray); early stage 2, mild parkinsonism (2, light gray); late stage 2’, mild parkinsonism (2’, light gray); stage 3, moderate parkinsonism (3, dark gray). Horizontal lines represent medians (IQR), except for Switch-Cost (mean ± SEM). *p < 0.05, coefficient estimates compared to BL and post hoc comparisons (Holm–Bonferroni corrected).

Before and during MPTP treatment, both monkeys performed a validated counter-demanding task discriminating motivational, cognitive, and motor behaviors^16^. On each trial, they could either perform a switching task (Work), where correct responses filled a gauge, or checked the gauge itself (Check) to monitor their progress toward a bonus reward delivered once full (Fig. 2A, see “Methods”). Motivation was assessed by the probability to check when the gauge was full (Reward Check); cognitive performance by the error rate on Switch trials (Error Switch) and Switch-Cost (response time difference between correct Switch and Non-Switch trials); and motor performance by response time on correct Non-Switch trials (mRT). Each monkey completed at least five sessions per stage (≥4 bonus rewards/session; Supplemental Table 1): M1 performed 10, 17, 13, 6, and 6 sessions at baseline, stage 1, stage 2, stage 2’, and stage 3, respectively; and M2 performed 10, 16, 9, 7, and 5 sessions.

During stage 1, just after starting MPTP treatment, no significant behavioral effects were found for both monkeys, consistent with an asymptomatic stage.

Stage 2 was the most prolonged stage, characterized by high variability in motor evaluations and inter-individual differences in DaT imaging. This led to a deeper behavioral analysis, which revealed that non-motor signs appeared in a specific chronology, distinguishing two substages. Early stage 2 affected motivation with decreased Check Reward (generalized linear mixed-effects model: *β* = −0.49 ± 0.06, *p* < 0.001, OR = 0.61), consistent in both monkeys (M1- stage 2: 21% [IQR 17–23] vs. BL: 28% [23–32]; M2- stage 2: 15% [14–16] vs. BL: 23% [21–27]) (Fig. 2B).

Subsequently, a late stage 2’ affected cognitive behavior with increased Switch Error (*β* = 0.58 ± 0.19, *p* = 0.011, OR = 1.78), observed in monkeys (M1- stage 2’: 6.4% [6.0–6.6] vs. BL: 2.8% [2.7–3.0]; M2- stage 2’: 8.1% [7.8–8.8] vs. BL: 6.0% [5.5–7]) (Fig. 2C). M2 also exhibited a negative Switch-Cost (BL: 45.1 ± 4.4 ms vs. stage 2’: −3.9 ± 5.7 ms), suggesting advanced cognitive impairment (Fig. 2D).

Finally, during stage 3, cognitive impairment deepened with a decreased Switch-Cost (*β* = −66.8 ± 13.3, *p* < 0.001, OR = −2.36) (Fig. 2D). Additionally, both monkeys exhibited slower motor response time with increased mRT (*β* = 0.15 ± 0.03, *p* < 0.001, OR = 1.17), consistently observed in both monkeys (M1- stage 3: 501 ms [460–603] vs. BL: 411 ms [372–478], M2- stage 3: 487 ms [399–676] vs. BL: 454 ms [390–536]) (Fig. 2E).

### Evolution of STN activity

STN local field potentials were recorded while monkeys performed the behavioral task at baseline and throughout MPTP treatment. Time-frequency representations of spectral power were averaged across trials within each condition and aligned to task events. Band-specific powers within defined time windows were then quantified and compared across stages.

**Fig. 3:**
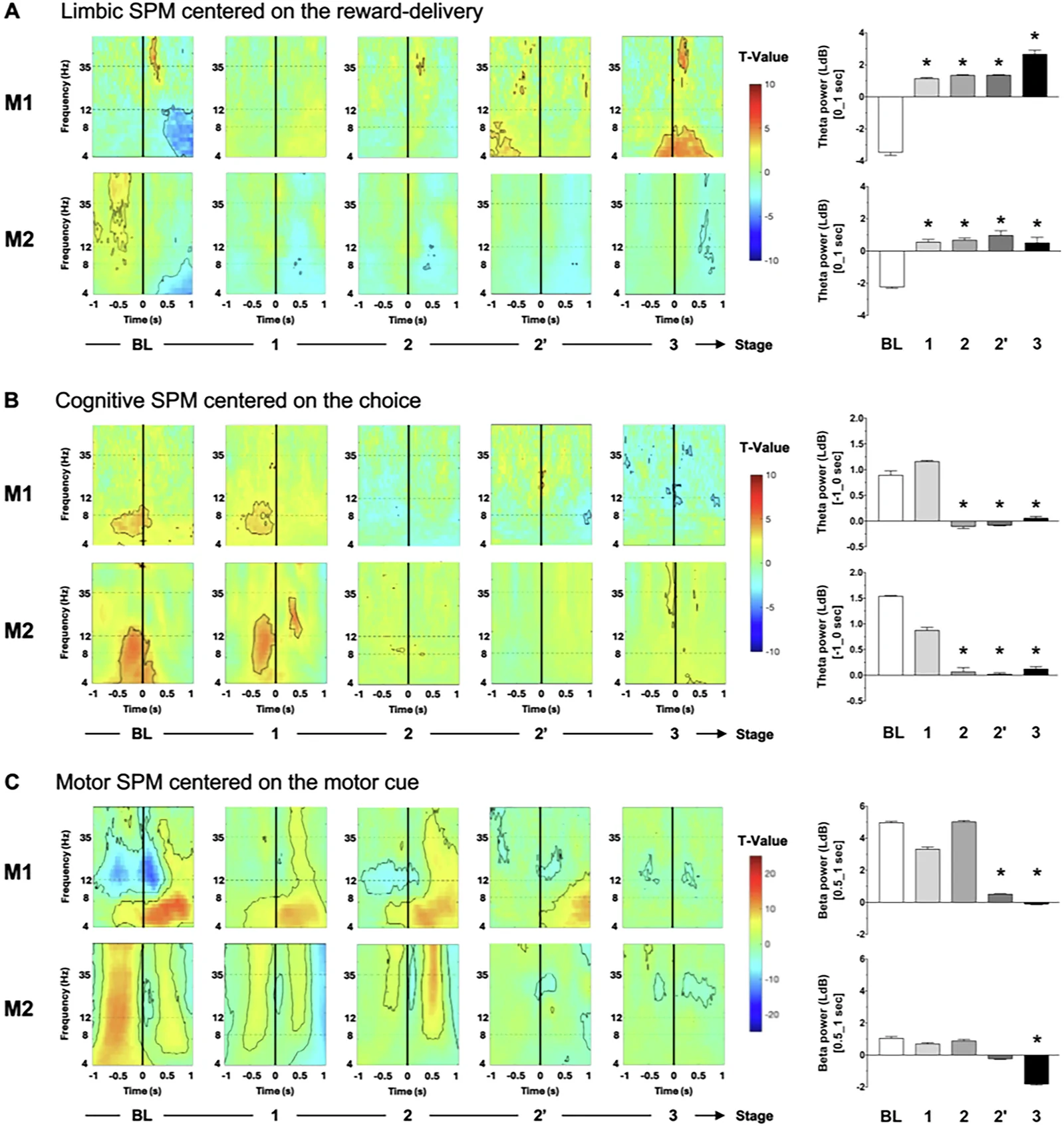
STN neuronal effect of MPTP treatment across stages. **A** Limbic statistical parametric maps (SPM) centered on the full gauge with reward delivery (vertical line at 0) relative to the gauge view without the reward, with theta quantification between 0 and 1 s. **B** Cognitive SPM centered on cognitive choice (vertical line at 0) to work relative to check with theta quantification between −1 and 0 s. **C** Motor SPM centered on the onset of Non-Switch stimulus (vertical line at 0) relative to a resting state, with beta quantification between 0.5 and 1 s. Stages: baseline, before MPTP treatment (BL); 1, asymptomatic; 2, early mild parkinsonism; 2’, late mild parkinsonism, 3, moderate parkinsonism. Significant values are encircled with solid lines, *p* < 0.05. Power quantifications are expressed in LdB with mean ± SEM, and *p < 0.05.

During reward delivery, baseline activity showed a significant decrease in theta oscillations (4–8 Hz) for both monkeys (*p* < 0.05, two-sample t-test) (Fig. 3A). This decreased theta disappeared, starting from stage 1, with theta power significantly higher at all parkinsonian stages compared to baseline (for **M1-** BL: −3.4 ± 0.2 LdB vs. stage 1: 1.3 ± 0.1 LdB, *p* < 0.001; for **M2-** BL: −2.2 ± 0.1 LdB vs. stage 1: 0.5 ± 0.2 LdB, *p* < 0.001).

When monkeys chose to work rather than check, baseline activity showed a significant increase in theta oscillations for both monkeys (*p* < 0.05, two-sample t-test) (Fig. 3B). This effect was preserved in stage 1 but disappeared from early stage 2, with theta power significantly lower than baseline (for **M1-** BL: 0.9 ± 0.1 LdB vs. stage 2: −0.1 ± 0.1 LdB, *p* < 0.001; for **M2-** BL: 1.5 ± 0.1 LdB vs. stage 2: 0.1 ± 0.1 LdB, *p* = 0.004).

At the onset of the motor cue, baseline activity showed a significant decrease in beta oscillations followed by a rebound beta increase for both monkeys (*p* < 0.05, one-sample t-test) (Fig. 3C). This rebound persisted during stage 1 and early stage 2 but disappeared from stage 2’. Quantification of beta rebound confirmed significant reductions at stage 2’ for M1 and at stage 3 for M2 (for **M1-** BL: 5.0 ± 0.1 LdB vs. stage 2’: 0.5 ± 0.3 LdB, *p* < 0.001; for **M2-** BL: 1.0 ± 0.1% vs. stage 3: −1.8 ± 0.1 LdB, *p* = 0.021).

### STN modulation on parkinsonian syndrome

In stage 3, when both monkeys exhibited a stable moderate parkinsonian syndrome, LFS (4 Hz) and HFS (130 Hz) were applied to the ventral and dorsal STN. Each animal completed at least five sessions per condition (≥4 bonus rewards/session; Supplemental Table 2). Despite inter-individual variability, consistent with PD patients’ reports^32^, generalized mixed-effects models showed converging effects.

**Fig. 4:**
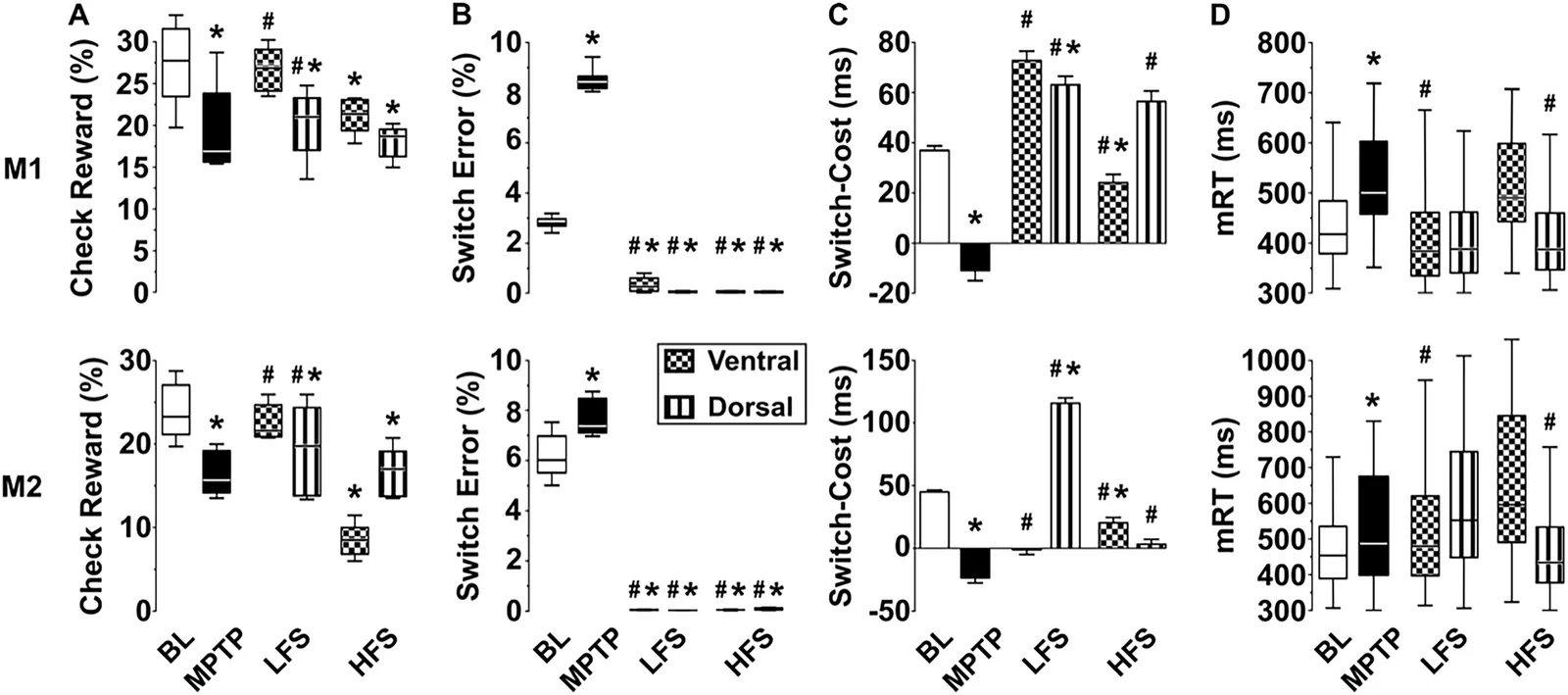
Behavioral effect of LFS (4 Hz) and HFS (130 Hz) on ventral (grid pattern) and dorsal (vertical lines) STN, on MPTP-treated monkeys. **A** Probabilities to check when the reward is available (Check Reward, %). **B** Errors on Switch trials (Switch Error, %). **C** Switch-Cost in ms, response time difference between Switch and Non-Switch trials. **D** Motor response time (mRT, ms). BL, baseline (white); MPTP, stage 3 (black); LFS, 4 Hz applied in stage 3; HFS, 130 Hz applied in stage 3. Horizontal lines represent medians (IQR), except for Switch-Cost (mean ± SEM). *p < 0.05 different from baseline and #*p* < 0.05 different from MPTP stage 3 off-stimulation, coefficient estimates, and post hoc comparisons (Holm–Bonferroni corrected).

Ventral LFS-STN restored the decreased motivation (Check Reward) to baseline value (compared to baseline: *β* = −0.05 ± 0.02, *p* = 0.762, OR = 0.95; compared to MPTP stage 3: *β* = 0.44 ± 0.03, *p* < 0.001, OR = 1.55), observed in both monkeys (**M1-** MPTP ventral LFS: 27% [IQR 24–27] vs. BL: 28% [24–32] and MPTP: 17% [16–24]; **M2-** MPTP ventral LFS: 22% [21–25] vs. BL: 23% [21–27] MPTP: 16% [14–19]) (Fig. 4A). Errors on Switch trials were almost eliminated with both LFS and HFS, regardless of dorsal or ventral STN stimulation, significantly different from baseline and MPTP (Fig. 4B).

In contrast, Switch-Cost increased with dorsal LFS STN compared to both baseline and MPTP (compared to baseline: *β* = 48.38 ± 15.81, *p* = 0.009, OR = 1.30; compared to MPTP stage 3: *β* = 115.24 ± 29.00, *p* < 0.001, OR = 3.09), observed in both monkeys (**M1-** MPTP dorsal LFS: 63.5 ± 3.4 vs. BL: 37.0 ± 1.8 and MPTP: −10.6 ± 4.1; **M2-** MPTP dorsal LFS: 116.0 ± 4.3 vs. BL: 45.1 ± 4.4 and MPTP: −40.3 ± 10.6) (Fig. 4C).

Finally, as expected dorsal HFS-STN restored the mRT to baseline value (compared to baseline: *β* = 0.04 ± 0.04, *p* = 0.279, OR = 1.04; compared to MPTP stage 3: *β* = −0.11, ±0.06, *p* = 0.042, OR = 0.90), for both monkeys (for **M1-** MPTP dorsal HFS: 388 ms [IQR 347–460], MPTP: 501 ms [460–603], and BL: 411 [372–478]; for **M2-** MPTP dorsal HFS: 435 ms [378–534], MPTP: 487 ms [399–676], and BL: 454 ms [390–536]). Interestingly, ventral LFS-STN also restored the mRT to baseline value (compared to baseline: *β* = 0.05 ± 0.08, *p* = 0.51, OR = 1.05; compared to MPTP stage 3: *β* = −0.11 ± 0.05, *p* = 0.036, OR = 0.90), for both monkeys (for **M1-** MPTP ventral LFS: 384 ms [335–461], MPTP: 501 ms [460–603], and BL: 411 [372–478]; for **M2-** MPTP ventral LFS: 480 ms [398–621], MPTP: 487 ms [399–676], and BL: 454 ms [390–536]) (Fig. 4D).

## Discussion

Using a progressive PD model, we demonstrate that non-motor impairments emerge prior to motor signs and are associated with early alterations in STN activity. Importantly, we identify a temporal sequence linking distinct oscillatory changes to specific behavioral impairments. In stable parkinsonism, we further show that ventral LFS selectively alleviates motivational deficits, while both ventral LFS and dorsal HFS improve motor impairments, highlighting the site- and frequency-specific contributions of STN circuits to both motor and non-motor outcomes.

Among NHP models, MPTP uniquely reproduces a comprehensive nigro-striatal dopamine deficit^17^, with human-like clinical features including motor symptoms, sleep/wake disturbances, and cognitive deficits^20,31,33^. In our progressive model, both monkeys developed a months-long worsening parkinsonian syndrome, mirroring the slow progression of human PD and allowing severity-stage discrimination consistent with previous studies. Interestingly, we observed a transient increase in striatal presynaptic dopamine reuptake for M1 during mild parkinsonism. Such fluctuations are consistent with preclinical and clinical observations^34–36^, where increased dopamine synthesis and turnover, as well as downregulation of DaT density, likely contribute to compensatory mechanisms during the prodromal stage.

We also observed CSF decreases in HVA, MHGP, and 5-HIAA, indicating impaired dopamine, noradrenergic, and serotonin neurotransmission, patterns reported across PD models and patients^37–39^, and correlating with movement disorders, depression, impulsivity, and cognitive impairments^37,40–42^.

**Fig. 5:**
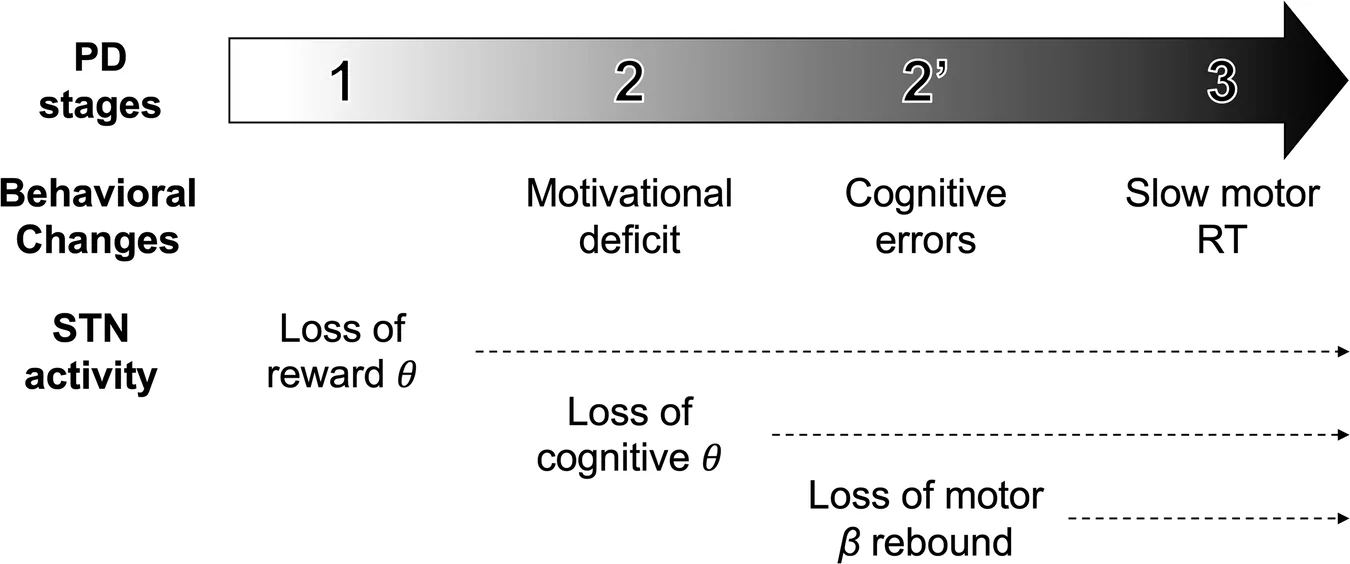
Study-based relationship between subthalamic nucleus (STN) activity alterations and the subsequent emergence of behavioral changes in a progressive model of Parkinson’s disease (PD). Chronic low-dose MPTP induced distinct stages: 1, asymptomatic; 2, mild parkinsonism with motivational deficits; 2’, novel intermediate stage with cognitive impairments; and 3: moderate parkinsonism with significant motor signs. RT, response time; *θ*, theta activity; *β*, beta activity.

In line with recent studies linking neural activity to PD symptoms severity^43–45^, we demonstrated that alterations in STN activity precede behavioral changes in a defined temporal sequence (motivational, followed by cognitive, and ultimately motor deficits), thereby defining parkinsonian stages (Fig. 5).

The first stage, after first MPTP injections, is characterized by the absence of significant behavioral changes and therefore considered asymptomatic. In parallel, we observed a baseline-related decrease in theta oscillations during reward delivery, which disappeared from this stage forward. STN theta activity has been associated with limbic processes: in parkinsonian rats as a predictor of addiction vulnerability^46^, and in PD patients with emotional voice processing^47^, reward and loss anticipation^12^, pathological gambling^48^, and depressive symptoms^13^. Notably, an EEG study reported that apathetic PD patients displayed higher baseline alpha-theta power than non-apathetic patients, suggesting that elevated alpha-theta activity may interfere with the neural dynamics required for incentivized movements^49^.

Early stage 2 was defined by reduced motivation, resembling apathy reported in PD models^50,51^and PD patients^52^. In parallel, decision-related STN theta activity disappeared from this stage onward, consistent with the role of prefrontal-STN circuits in deliberative action selection during a conflict state^11,53^. Additionally, studies have shown that increased STN theta-alpha power is associated with decision-making and conflict resolution^54,55^, whereas a decrease is associated with impulse control deficits and obsessive-compulsive disorder^56,57^.

Late stage 2’ was characterized by cognitive deficits resembling PD mild cognitive impairment^58–60^. Interestingly, both error rate and switch-cost were impacted for M2, whereas only error rate increased for M1, consistent with the transient striatal DaT increase and potential compensatory mechanisms previously discussed. In parallel, movement-related beta dynamics changed: the characteristic movement pattern of beta desynchronization followed by a rebound^61^ was lost from this stage onward. Increased STN beta activity is a validated biomarker in PD and scales with motor symptoms severity^10,62–66^. Motor beta activity variations could thus be flattened by the overall beta increase as parkinsonism progresses.

In stage 3, in addition to motivational and cognitive deficits, clear motor manifestations appeared. These observations are consistent with the course of PD, in which non-motor symptoms precede motor symptoms by several years^2,42^.

This study has several limitations that should be considered. STN-DBS remains the main symptomatic surgical treatment in PD, typically with HFS (130 Hz) targeting dorsolateral STN^7^. Alternative paradigms have been investigated, with dorsal LFS (4–5 Hz) showing promising cognitive improvement^24,25^. Based on these findings, we tested both HFS and LFS in dorsal and ventral STN. Despite inter-individual variability, consistent with clinical reports^27,32^, we observed converging site and frequency-specific effects.

As expected, HFS in dorsal STN improved motor impairments. However, when applied in ventral STN, it aggravated motivational deficits for M2, mirroring the exacerbation of apathy and mood disturbances reported in some PD patients after DBS surgery^22^.

Conversely, LFS in dorsal STN increased the Switch-Cost, consistent with previous 4 Hz STN-DBS findings showing improved inhibition of impulsive/premature responses by engaging prefrontal-STN circuits^24^.

Most strikingly, we found for the first time that ventral LFS restored parkinsonian motivational deficits to baseline levels. This effect can be interpreted by functional organization of the STN, where the ventromedial territory is preferentially connected to limbic circuits^6,67,68^. Ventral LFS could thus modulate the ventromedial prefrontal-STN hyperdirect pathway, potentially restoring physiological oscillatory dynamics^24^. Indeed, theta and low-alpha oscillations in the STN have been associated with motivational and decision-making processes, their disruption being directly correlated with apathy and neuropsychiatric symptoms in PD patients^69,70^. Ventral LFS may thus restore functional low-frequency activity within the limbic STN, thereby facilitating the engagement of motivational circuits. This interpretation is consistent with clinical observations showing that stimulation closer to the ventral STN is associated with improvements in anxiety and depressive symptoms^66^, although 4 Hz stimulation in this region has not yet been explored^71^.

Finally, cognitive errors were markedly reduced regardless of stimulation location and frequency. This robust effect is unlikely to be solely explained by practice or learning, as error rates increased with the progression of the parkinsonian state (stages 2’ and 3), despite extensive task exposure. A potential contribution of generalized arousal cannot be excluded, although increased arousal does not systematically translate into improved performance and depends on task characteristics^72^.

This study has several limitations that should be considered. While our parkinsonian model offers strong translational value by reproducing a progressive syndrome with both motor and non-motor clinical features, the underlying degenerative mechanisms differ between MPTP toxicity and human PD. Numerous studies have discussed strengths and limitations of this model, most notably the lack of Lewy bodies, a hallmark of alpha-synucleinopathies^17,73–75^.

Another limitation is the small sample size, inherent to NHP research, where ethical and logistical constraints preclude large cohorts^76,77^. Nevertheless, intra-individual longitudinal comparisons across parkinsonian stages are a strength of preclinical studies, inaccessible in human research. Furthermore, variability in STN recording and stimulation sites between the two monkeys may have contributed to inter-individual discrepancies, similar to clinical DBS where subtle differences in lead location strongly influence behavioral outcomes^78,79^.

Finally, it is important to acknowledge that we used task-derived behavioral phenotypes to define the transition between stage 2 and stage 2’. While this functional staging approach could introduce potential circularity regarding the causal role of STN activity, we argue that these behavioral changes represent the manifestation of an underlying, continuous neurophysiological drift initiated during the preceding, clinically silent periods. Our results thus highlight a temporal evolution of circuit dysfunction, where STN electrophysiological alterations precede the manifestation of behavioral deficits.

Our findings demonstrate that progressive dopamine depletion reshapes STN functional activity in a way that temporally precedes the emergence of non-motor and motor manifestations. Motivational and cognitive impairments should therefore be expected early in PD and actively assessed with appropriate clinical evaluations. Furthermore, the site and frequency-specific effects of ventral LFS and dorsal HFS highlight the therapeutic potential of adaptive DBS strategies, offering a promising approach to manage both motor and non-motor symptoms in PD.

## Methods

### Animals

This study involved two 8-year-old monkeys (*Macaca fascicularis*, CRP, Port Louis, Mauritius), one male (M1, 9 kg) and one female (M2, 4 kg). They were pair-housed in a temperature- (22 ± 1 °C) and humidity-controlled (50 ± 5%) facility with a 12 h light–dark cycle. Animals had ad libitum access to primate chow and water, and were provided with supplemental fruits once daily. All experimental procedures were conducted in accordance with the European Communities Council Directive (2010/63/EU) for the care and use of laboratory animals and complied with the recommendations of the French National Committee (2013/113). All protocols were approved by the local Ethical Committee (#04; authorization n°2019013116115695). All procedures were designed to minimize animal suffering and to reduce the number of animals used. Animals were monitored daily by trained staff, and their health and welfare were closely supervised throughout the study. All procedures complied with ARRIVE guidelines.

### Randomization and blinding

Given the limited number of animals (*n* = 2), formal randomization was not applicable. However, experimental conditions were balanced across sessions. Investigators were not blinded during data collection, but analyses were performed using automated and standardized procedures to minimize potential bias.

### Experimental design

Monkeys performed a counter-demanding task assessing limbic, cognitive, and motor behaviors, as previously described^16^. Animals were habituated to a primate chair (Crist Instrument Co., MD, USA) and interacted with a touch screen (Elo Touch Solutions, Inc.).

### Behavioral task

In each trial, they chose either to Work on rewarded switching task (triangle, upper center) or to Check their progress with a gauge indicating the proximity of a bonus reward (cross, bottom center). When selecting Work, a blue square (Non-Switch) or an orange star (Switch) appeared randomly left or right, and monkeys had to touch the blue square directly or the opposite side of the orange star. Non-Switch trials occurred with higher probability (80%) than Switch trials (20%). Correct responses within 2000 ms were rewarded (sweet liquid, 1 ml) by a computer-controlled system (Crist Instrument Co., MD, USA), and each trial ended with a 1000 ms red circle before the next Work/Check choice. In Check trials, a gauge (large green circle) was displayed for 5000 ms, filling proportionally with correct Work responses (randomly 8, 16, 24, or 32 required). When Checking a full gauge, a bonus reward (sweet liquid, 5 ml) was delivered, after which the gauge reset. Incorrect responses did not affect the gauge.

### Behavioral analyses

Only sessions with more than four earned bonus rewards were included. Response times (RT, time between the appearance and touch of the stimulus) were measured from correct trials: motor RT (mRT, motor behavior) for Non-Switch trials and cognitive RT (cRT) for Switch trials. Differences between cRT and mRT represented the Switch-Cost (cognitive behavior). Error rate on Switch trials (cognitive behavior) and probabilities to Check when the gauge was full (Reward Check, limbic behavior) were calculated.

### MPTP treatment

Monkeys were chronically intoxicated with biweekly intramuscular injections of 1-methyl-4-phenyl-1,2,3,6-tetrahydropyridine (MPTP; 0.2–0.5 mg/kg, i.m. diluted in NaCl 0.9%) under light anesthesia induced by ketamine (2–4 mg/kg, i.m.), as previously described^31^. After each injection, motor score was assessed, and doses were adjusted. M1 received 20 injections over 43 weeks (5.45 mg/kg total), and M2 received 18 injections over 39 weeks (6.7 mg/kg total). Both exhibited a similar stable parkinsonian syndrome.

### Motor score

Behavioral evaluation was performed throughout MPTP treatment in the home cage, using a validated macaque parkinsonism scale^31,80^. Eight criteria were scored from 0 (no impairment) to 3 (maximal impairment), including each arm movement frequency, general activity, posture, bradykinesia, tremor, feeding, freezing and vocalizations (Supplemental Fig. 1). According to the literature, three severity stages were identified: stage 1, asymptomatic (0–3); stage 2, mild parkinsonism (4–10); and stage 3, moderate parkinsonism without care needed (>10)^81^.

### DaT SPECT imaging

Striatal dopaminergic system was assessed at baseline and across parkinsonian stages using Ioflupane tracer (I123, 90 MBq), which binds to presynaptic dopamine transporters (DaT). For all exams, monkeys were anesthetized for intravenous tracer injection, and images were acquired 3 h later. A zoom lens was applied for M2 due to sexual dimorphism. Semi-quantitative analysis of striatal ROI was calculated from the three slices with maximal radioactivity and normalized to an occipital cortex ROI.

### CSF analysis

At baseline and throughout MPTP treatment, CSF was collected under anesthesia by lumbar puncture (L4/L5) using a 22 g Quincke spinal needle (BD^TM^). Samples were stored on ice in sterile 1.5 mL polypropylene tubes and frozen at −80 °C. Monoaminergic metabolites (homovanillic acid, HVA; 5-hydroxyindole acetic acid, 5-HIAA; 3-methoxy-4-hydroxyphenylglycol, MHPG; and 3-orthomethyl-DOPA, 3-OMD) were quantified by ion pair high-performance liquid chromatography with electrochemical detection, as previously described^82^.

### Surgical procedure

Electrode implantation was performed under aseptic conditions and general anesthesia, as previously described^16^. Monkeys were initially anesthetized with ketamine (7 mg/kg, i.m.) and xylazine (0.6 mg/kg, i.m.), and maintained anesthetized with isoflurane. Lidocaine 1% was used for local anesthesia. Saline solution (NaCl 0.9%, Sigma-Aldrich) was infused intravenously during surgery for drug access and hydration. Anesthesia depth and physiological status were continuously monitored throughout the procedure using vital parameters, including oxygen saturation and respiratory rate, and adjusted as needed to maintain stable anesthesia.

Pre-operative 7 T MRI (IRMaGE, Grenoble) allowed the STN identification and per-operative X-rays with ventriculography localized the anterior and posterior commissures (ACPC), with 2 ml injection of contrast agent (Iopamiron 200, iodine 200 mg/ml, Bracc) into the left lateral ventricle. STN borders were refined using micro-recordings (250 µm diameter, 0.8–1.2 MOhm, FHC), based on firing rate and pattern. The right STN was implanted with a quadripolar DBS electrode (0.8 mm diameter, four 0.5 mm contacts in two rows, 0.5 mm apart, and two columns; Heraus©, USA), allowing recordings and two-level stimulations. Electrode was placed at the following stereotactic coordinates: anterior 6/12th of ACPC, 4–5 mm from the midline, with deepest contacts at the lower STN border. The implantation was confirmed by radiography and ventriculography (see ref. ^16^, Fig. 1D, E), and the reference fixed at the left occipital level. A stainless-steel head holder (Crist Instrument Co., MD, USA) was placed at the back of the skull to maintain the head. Post-operative analgesic/anti-inflammatory therapy (Ketoprofen, 2 mg/kg, i.m.) was administered for 1 week.

### STN recording

Baseline recordings began 2 weeks after surgery and continue throughout MPTP treatment, using a common occipital reference and a multichannel system (AlphaOmega Engineering, Israel). Signals were sampled at 1375 Hz, aligned to task events (±1000 ms), and analyzed using MATLAB and ImaGIN toolbox (MathWorks). Time-frequency representations of spectral power (2–200 Hz) were computed using a multitaper sliding window with orthogonal discrete prolate Slepian spheroidal (DPSS) tapers, adapted to the frequency and sliding-window length. Single-trial power spectra were averaged by condition and normalized to a pre-stimulus baseline (−1000 to −250 ms) using the formula ${{\rm{L}}}_{{\rm{dB}}}=10{\log }_{10}\left({\rm{P}}/{\rm{B}}\right)$, where P is peristimulus power and B is baseline power. Data are expressed in LdB (mean ± SEM) for specific frequency bands defined as theta (4–8 Hz), alpha (8–12 Hz), beta (12–35 Hz), and gamma (35–200 Hz).

### STN stimulation

After MPTP treatment, continuous bipolar stimulation was applied in ventral or dorsal STN, at low frequency stimulation (LFS, 4 Hz), shown to improve cognitive deficits in PD patients^24,25^, and high-frequency stimulation (HFS, 130 Hz), clinical standard for PD motor symptoms^83^. For each frequency, stimulation ranges were conducted until side effects occurred (e.g., ipsilateral monocular deviation, head rotation, lip contraction), and intensities set at 80% of this threshold (0.08–0.2 mA; pulse width 60 µs). The number of trials per condition is detailed in Supplementary Table 2.

### Statistical analyses of behavioral data

Analyses were conducted on MATLAB (The MathWorks Inc.) using generalized linear mixed-effects models (GLMMs) to account for the hierarchical structure of the data (sessions nested within subjects) and inter-individual variability. A linear predictor (*X*) was defined to account for the fixed effects of Stage and Session, and random variations between subjects:

1 $$X={\beta }_{0}+{\beta }_{{condition}}{\rm{\cdot }}{condition}+{\beta }_{{session}}{\rm{\cdot }}{session}+\left(1+{condition}/{subject}\right)$$

where *β*_0_ is the intercept, condition is either MPTP stages (BL, 1, 2, 2’, 3) or stimulation conditions (ventral LFS, dorsal LFS, ventral HFS, dorsal HFS) and session is the mean-centered session index (to control for time-related effects such as task repetition or fatigue). The random-effects structure includes random intercepts and random slopes for stages per subject. Appropriate link functions were applied to *X* for each variable as follows:

Check Reward & Switch Error: a binomial distribution with a logit link function.

Switch-Cost: a normal distribution with an identity link function, after normality tests.

mRT: a gamma distribution with a log link function, to account for the right-skewed distribution of response time and the inherent variance-mean relationship.

Models were fitted using Maximum Likelihood estimation (Laplace approximation). Statistical significance for fixed effects was determined based on the model’s coefficient estimates, followed by multiple comparisons with Bonferroni-Holm correction. Results are reported as fixed-effects estimated coefficients (*β*) ± standard errors (SE), *p* values, and odds ratios (OR, as exponentiated fixed-effects coefficients, *e*^*β*^) and Cohen’s d for Switch-Cost (*β*/*σ*total). Unless otherwise stated, data are presented as median [IQR], except for Switch-Cost (mean ± SEM). Statistical significance was defined at *p* < 0.05.

### Statistical parametric maps

Post-hoc t-tests for time-frequency representations of spectral power were Bonferroni corrected, with “one-sample” t-test to compare signal of one event, and “two-sample” t-test to discriminate significant differences between two events (T-Values). These analyses were plotted as statistical parametric maps (SPM) with significant value encircled (*p* < 0.05). For band-specific analyses, mean relative power were compared across stages using ANOVA followed by Tukey’s test. Data are presented as mean ± SEM, and a difference was considered statistically significant for a value of *p* < 0.05.

### End of study

At the end of the study, animals were deeply anesthetized with ketamine (10 mg/kg, i.m.) and pentobarbital (25 mg/kg, i.m.), and transcardially perfused with 0.9% saline followed by 4% paraformaldehyde in 0.1 M phosphate buffer (pH 7.4), in accordance with institutional and national guidelines. Brains were then collected for post-mortem analyses.

## Supplementary information

Supplementary Information

## Data Availability

The datasets generated and/or analyzed during the current study are not publicly available due to ethical restrictions related to non-human primate research, but are available from the corresponding author on reasonable request. The code used for the analyses in this study is available from the corresponding author on reasonable request.
