# Supplementary material for: Behavioral changes preceded by subthalamic nucleus activity alterations in a progressive macaque model of Parkinson’s disease
Source: NPJ Parkinsons Dis. 2026 Jul 31;12:242. doi: 10.1038/s41531-026-01498-2 (PMC13638992; doi:10.1038/s41531-026-01498-2)
Supplement: Supplementary file 1 [file 41531_2026_1498_MOESM1_ESM.pdf]

Supplemental Table 1

		**BL**		**1**		**2**		**2'**		**3**	
		n (trials)	median (IQR)	n	median (IQR)	n	median (IQR)	n	median (IQR)	n	median (IQR)
**M1**	Check Reward (%)	2013	27.8 (23.6-31.6)	1251	27.3 (21.9-28.7)	1312	21.2 (17.2-22.6)	312	16.0 (15.0-21.0)	194	17.0 (15.7-23.9)
	Switch Error (%)	943	2.8 (2.7-3.0)	695	4.5 (4.3-4.8)	998	2.7 (2.3-3.0)	284	6.4 (6.1-6.6)	173	8.4 (8.2-8.7)
	Switch-Cost (ms) - mean ± SEM<br>n sessions	10 sessions	37.0 ± 1.8	17	68. 2 ± 2.6	13	16.2 ± 2.1	6	38.7 ± 5.6	6	-10.6 ± 4.1
	mRT (ms)	3992	411 (372-478)	2816	419 (374-488)	4158	419 (362-496)	1176	356 (311-423)	685	501 (460-603)
**M2**	Check Reward (%)	1082	23.3 (21.2-27.1)	894	20.2 (17.2-21.3)	170	14.9 (14.1-16.0)	110	17.7 (15.6-19.6)	82	15.7 (14.2-19.2)
	Switch Error (%)	772	6.0 (5.5-7.0)	771	3.6 (3.3-4.0)	209	2.0 (1.3-2.5)	103	8.1 (7.8-8.8)	101	7.4 (7.1-8.5)
	Switch-Cost (ms) - mean ± SEM<br>n sessions	10 sessions	45.1 ± 4.4	16	21.0 ± 3.0	9	7.5 ± 2.4	7	-3.9 ± 5.7	5	-40.3 ± 10.6
	mRT (ms)	3084	454 (390-536)	3072	408 (371-471)	812	411 (364-485)	452	380 (335-483)	461	487 (399-676)

Supplemental Table 2

		BL		3		4_Ventral		4_Dorsal		130_Ventral		130_Dorsal	
		n (trials)	median (IQR)	n	median (IQR)	n	median (IQR)	n	median (IQR)	n	median (IQR)	n	median (IQR)
**M1**	Check Reward (%)	2013	27.8 (23.6-31.6)	194	17.0 (15.7-23.9)	362	26.9 (24.2-29.1)	360	21.1 (17.1-23.3)	229	21.4 (19.4-23.1)	413	18.8 (16.3-19.6
	Switch Error (%)	943	2.8 (2.7-3.0)	173	8.4 (8.2-8.7)	291	0.28 (0.09-0.65)	221	0.06 (0.04-0.08)	165	0.07 (0.03-0.09)	298	0.06 (0.03-0.07)
	Switch-Cost (ms) - mean ± SEM<br>n sessions	10 sessions	37.0 ± 1.8	6	-10.6 ± 4.1	7	73.1 ± 3.8	7	63.5 ± 3.4	6	24.3 ± 3.3	9	56.8 ± 4.1
	mRT (ms)	3992	411 (372-478)	685	501 (460-603)	1239	384 (335-461)	968	388 (341-362)	756	490 (441-600)	1204	388 (347-460)
**M2**	Check Reward (%)	1082	23.3 (21.2-27.1)	82	15.7 (14.2-19.2)	63	21.6 (20.9-24.7)	77	19.8 (13.8-24.4)	47	8.5 (6.8-10.0)	82	17.0 (13.8-19.1)
	Switch Error (%)	772	6.0 (5.5-7.0)	101	7.4 (7.1-8.5)	61	0.04 (0.04-0.05)	50	0.07 (0.04-0.08)	53	0.14 (0.11-0.15)	73	0.06 (0.06-0.08)
	Switch-Cost (ms) - mean ± SEM<br>n sessions	10 sessions	45.1 ± 4.4	5	-40.3 ± 10.6	5	-1.1 ± 3.8	5	116.0 ± 4.3	5	20.5 ± 4.2	6	3.3 ± 3.9
	mRT (ms)	3084	454 (390-536)	461	487 (399-676)	205	480 (398-621)	153	553 (449-745)	178	597 (491-845)	236	435 (378-534)

Date: …………………….

MPTP n° …….
Dose: …………….
Dose cumulative:…..

Frequency of arm movements (for each arm)	General activity
0= no difference with control	0= no difference with control
1= frequent	1= frequent
2= sometimes	2= sometimes
3= none	3= none

Posture	Bradykinesia
0= normal rising position	0= no difference with control
1= flexed posture (0°<F<45°)	1= mild slowing of overall movements
2= severely flexed posture (90°<F)	2= severe slowing of movements
3= dystonic posture	3= akinesia (no movements)

Tremor	Eating
0= none	0= normal eating
1= some episodes	1= slight decrease
2= frequent episodes	2= severe decrease
3= persistent	3= no eating alone

Freezing	Vocalization
0= none	0= frequent
1= some episodes	1= sometimes
2= frequent episodes	2= none

Supplemental Figure 1 : Rating scale used to assess the parkinsonian state in MPTP-treated non-human primates.
